# The combination of intravenous Reolysin and gemcitabine induces reovirus replication and endoplasmic reticular stress in a patient with KRAS-activated pancreatic cancer

**DOI:** 10.1186/s12885-015-1518-0

**Published:** 2015-07-10

**Authors:** Devalingam Mahalingam, Sukeshi Patel, Gerard Nuovo, George Gill, Giovanni Selvaggi, Matt Coffey, Steffan T. Nawrocki

**Affiliations:** 1 Division of Hematology/Oncology, Cancer Therapy and Research Center at The University of Texas Health Science Center at San Antonio, 7979 Wurzbach Rd, San Antonio, TX 78229 USA; 2 Comprehensive Cancer Center, Ohio State University, Columbus, OH USA; 3 Oncolytics Biotech, Inc., Calgary, AB Canada

**Keywords:** Reolysin, Reovirus, Oncolytic virus, RAS, Pancreatic cancer, Gemcitabine, ER stress, NOXA

## Abstract

**Background:**

Activating mutations in RAS are present in the majority of pancreatic cancer cases and represent an ideal therapeutic target. Reolysin is a proprietary formulation of oncolytic reovirus that is currently being evaluated in multiple clinical trials due to its ability to selectively replicate in cells harboring an activated RAS pathway. Here we report for the first time the presence of reovirus replication and induction of endoplasmic reticular (ER) stress in a primary tumor specimen collected from a pancreatic cancer patient receiving intravenous Reolysin and gemcitabine.

**Case presentation:**

We describe the case of a 54-year old patient diagnosed with pancreatic adenocarcinoma in February 2012. Analysis of a tumor biopsy revealed an activating KRAS mutation (G12D) and the patient was started on first-line treatment with Reolysin in combination with gemcitabine in March 2012. Stable disease was achieved with significant improvement in cancer-related pain. Following 25 cycles of treatment over 23 months, a second biopsy was collected and immunohistochemical analyses revealed the presence of reovirus replication and induction of the ER stress-related gene GRP78/BIP and the pro-apoptotic protein NOXA. Importantly, co-localization of reoviral protein and active caspase-3 was also observed in the biopsy specimen.

**Conclusion:**

This is the first report of reoviral protein detection in primary tumor biopsies taken from a pancreatic cancer patient receiving intravenous Reolysin therapy. The accumulation of reoviral protein was associated with ER stress induction and caspase-3 processing suggesting that Reolysin and gemcitabine treatment exhibited direct pro-apoptotic activity against the tumor.

## Background

Reolysin is a formulation of wild-type oncolytic reovirus that is currently under investigation in multiple randomized phase II clinical trials in solid tumors, including combination with taxane-based regimens for the treatment of patients with head and neck carcinoma, non-small cell lung cancer (NSCLC), prostate cancer, pancreatic cancer, and ovarian cancer [1–5]. Reoviruses have been reported to selectively replicate in cancer cells harboring an activated RAS pathway [6]. The preferential replication of reovirus in cells with activated RAS is due to RAS’s ability to inhibit double-stranded RNA-activated protein kinase (PKR), a key sensor that recognizes viral particles and results in abrogation of protein synthesis by phosphorylation of eukaryotic initiation factor 2 alpha (eif2α) [7]. Failure to activate PKR allows viral replication to continue unchecked in RAS-activated cells. Since RAS mutations are present in most patients with pancreatic cancer, Reolysin has been studied against this tumor type [8]. Reolysin has demonstrated promising activity in preclinical models of pancreatic cancer [9, 10]. A previous Phase I study established the safety of the combination of intravenous Reolysin with gemcitabine [4]. Subsequently, a Phase II study at our institution demonstrated a significant improvement in clinical benefit in response to Reolysin in combination with gemcitabine in patients with advanced pancreatic cancer (manuscript in preparation). However, given that pancreatic cancers are hypovascular tumors surrounded by dense desmoplastic tissue, it is postulated that chemotherapeutic agents often fail to reach the primary tumor. Therefore, it is essential to assess drug or target delivery into primary tumors and yet the identification of reovirus in primary tumor specimens and validation of biomarkers of clinical activity have not been investigated in pancreatic cancer. Here we report the first evidence of active reovirus replication in a primary tumor sample taken from a pancreatic cancer patient enrolled in the Phase II trial of intravenous Reolysin and gemcitabine. We also show induction of endoplasmic reticular (ER) stress, the pro-apoptotic BH3-only family member NOXA, and activation of caspase-3 in pancreatic tumor biopsies post-treatment.

## Case presentation

The patient is a 54-year-old gentleman, who presented with a few months of mid-epigastric pain, nausea and vomiting with associated weight loss in February 2012. CT and MRI scans revealed a 3.3 × 3.1 cm pancreatic head mass encasing superior mesenteric artery and vein with associated mesenteric periportal lymphadenopathy. He also had sub-centimeter lung nodules presumed to be metastatic deposits. He thus had a clinical stage 4 unresectable pancreatic cancer. Genomic analysis of tumor biopsies revealed the presence of KRAS mutation (G12D) and loss of CDKN2A/B.

**Fig. 1:**
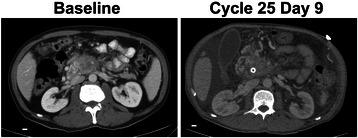
CT scans of pancreatic cancer patient. CT scan imaging at baseline (left) and at time of biopsy (right) demonstrates stability of hypovascular pancreatic mass. White bar indicates 1 cm

The patient was placed on a clinical trial with first-line treatment of Reolysin and gemcitabine, receiving cycle one day one on March 2012. Reolysin was administered at a dose of 1 × 10^10^ TCID_50_ IV on days 1, 2, 8, and 9 (immediately after gemcitabine on days 1 and 8) in combination with 800 mg/m^2^ IV gemcitabine on days 1 and 8, with 21-day cycles. The patient displayed a clinical response with improvement in cancer-related pain. The best radiographical response was documented as stable disease by Response Evaluation Criteria in Solid Tumors (RECIST) guidelines (Fig. 1) [11].

**Fig. 2:**
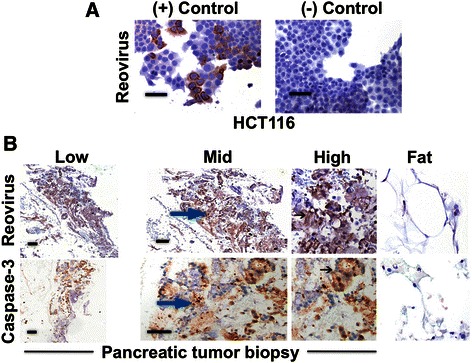
Reoviral protein accumulation and active caspase-3 levels in tumor biopsies. **a** Reovirus replicates in the HCT116 colon cancer cell line. HCT116 cells treated or untreated with Reolysin served as a positive and negative control for reoviral protein IHC. Brown staining indicates positive staining. **b** Detection of reoviral and active caspase-3 protein in a biopsy specimen obtained from a pancreatic cancer patient following Reolysin and gemcitabine therapy. Reoviral and active caspase-3 levels were detected by IHC. Low, middle, and high magnification images are displayed. Arrows denote positive brown staining representing positive reoviral and active caspase-3 staining, respectively. IHC of fat cells (far right) from the same pancreatic cancer patient tissue sample served as an internal negative control. Bar represents 75 microns. The staining score for both reoviral and active caspase-3 protein was 3+

**Fig. 3:**
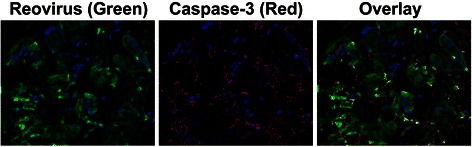
Co-expression of reoviral protein and caspase-3 is consistent with productive lytic infection in the patient’s pancreatic cancer cells when treated with intravenous Reolysin and gemcitabine. Blue indicates the nucleus of the cancer cell. Fluorescent green is the reoviral protein and fluorescent red is active caspase-3 protein. Yellow represents co-localization of reovirus and active caspase-3 in the same cancer cells after co-expression IHC analysis. The Nuance system converts each signal to a fluorescent-based signal to determine co-expression of the two targets of interest

**Fig. 4:**
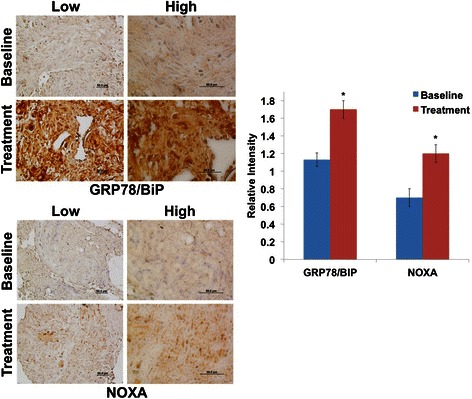
IHC analysis of GRP78/BiP and NOXA expression in biopsy specimens taken prior to (baseline) and following treatment with Reolysin and gemcitabine. IHC was performed on paraffin-embedded tumor sections followed by heat-induced epitope retrieval. (Left) GRP78/BiP, a marker of ER stress induction, is induced following treatment. NOXA, a pro-apoptotic gene, is also significantly increased after treatment. (Right) Quantification of the relative intensity of IHC staining in the biopsy specimens was performed using ImageJ software. Mean ± SD, n = 3. *Indicates a significant difference compared to baseline, *p* < 0.05

With the patient on treatment, a biopsy of the pancreatic mass was performed after cycle 25 day 8 in February 2014. The biopsy features were consistent with the diagnosis of pancreatic adenocarcinoma, with confirmed KRAS mutation (G12D) and loss of CDKN2A/B. Immunohistochemistry (IHC) was performed on Reolysin-treated or untreated HCT116 colon cancer cells as a positive and negative control for reovirus staining, respectively (Fig. 2a). Viral replication was detected using antibodies against the reovirus protein, as the presence of viral RNA may not necessarily imply infectious virus particles. A polyclonal antibody, raised in goats, was derived from mature reovirus viral capsid proteins [12]. Importantly, IHC analyses of biopsy specimens from a pancreatic cancer patient revealed strong positivity for reoviral protein and activated caspase 3 within the tumor (Fig. 2b). Biopsies from pancreatic cancer patients frequently contain benign fat, which may serve as an excellent internal negative control. Images of the stained fat cells were negative for reovirus and active caspase-3 and were from the same tissues that displayed positive staining for reovirus and active caspase-3 (Fig. 2b). Serial section analysis showed a very high concordance of reoviral protein and activated caspase-3, which is characteristic of a productive reovirus infection. In addition, co-expression analysis demonstrated that the reoviral protein and active caspase-3 were being expressed in many of the same cancer cells (Fig. 3). Our preclinical studies with Reolysin identified induction of ER stress and NOXA to be key determinants for Reolysin-mediated apoptosis [9, 13]. In agreement with the induction of active caspase-3, we also noted a significant increase in the expression of GRP78/BIP, which is commonly induced following ER stress and NOXA in the biopsy sample following Reolysin and gemcitabine treatment (Fig. 4).

Toxicities were manageable and included grade 1 fever likely due to Reolysin and grade 3 thrombocytopenia and neutropenia due to gemcitabine. The patient also had a biliary obstruction, which required stenting in November 2013. He completed 27 cycles of treatment with the last one in April 2014. At this time, he presented with disease progression with ascites and jaundice.

## Conclusions

To our knowledge, this is the first report that identified reoviral protein within a primary pancreatic tumor following systemic Reolysin therapy. These findings demonstrate that Reolysin and gemcitabine treatment may result in reovirus replication, ER stress, and apoptosis in patients treated with this regimen. Preliminary results from the Phase II study at our institution for patients with advanced pancreatic cancer treated with gemcitabine and Reolysin showed a clinical benefit with acceptable tolerability. Although this patient did not have tumor shrinkage, the patient did have prolonged stable disease for more than 2 years as well as symptomatic improvement with decreased cancer-related pain.

Since pancreatic cancers are hypovascular tumors surrounded by dense desmoplastic tissue, drug resistance may occur as chemotherapeutic agents fail to penetrate the primary tumor. This case establishes Reolysin’s ability to invade the dense desmoplastic tissue surrounding the hypovascular pancreatic cancer, suggesting that Reolysin may be an effective agent against drug resistant tumors. Since the majority of pancreatic cancers have RAS pathway activation, Reolysin serves as a potential promising active agent to overcome this barrier when combined with conventional chemotherapy [8].

The mechanisms of Reolysin’s anticancer activity are pleiotrophic and have been reported to include tumor lysis, ER stress, apoptosis, and stimulation of an immune response against the tumor in preclinical models [13–17]. Here we provide evidence in the clinical setting that the combination of Reolysin and gemcitabine displays direct anticancer effects against the primary tumor. The multitude of mechanisms by which Reolysin attacks tumor cells is an advantage that may be able to help overcome drug resistance and contribute to its ability to augment the activity of multiple conventional chemotherapeutic agents.

We have also shown significant induction of the ER stress associated markers GRP78/BIP and NOXA in the biopsy specimen analyzed following Reolysin and gemcitabine treatment. Our preclinical studies determined that induction of ER stress and the BH3-only pro-apoptotic gene NOXA are key mediators of Reolysin-induced apoptosis [9, 13]. Since cells with activated RAS are frequently under constitutive ER stress, further induction of this stress response may result in reaching a threshold where apoptosis is initiated. Additional studies combining Reolysin with other agents that trigger ER stress (i.e. bortezomib) may yield enhanced anticancer activity.

Taken together, we are the first to demonstrate reoviral protein accumulation in a primary tumor from a cancer patient treated with systemic Reolysin and gemcitabine. This treatment regimen yielded stable disease for more than 2 years in a patient with advanced pancreatic cancer, which is a significant improvement compared to the historical median survival of 6–12 months in this patient population [18, 19].

### Consent

Written informed consent was obtained from the patient for publication of this case report and accompanying images. A copy of the written consent is available for review by the Editor of this journal.

## Abbreviations

ER: Endoplasmic reticulum
PKR: Double-stranded RNA-activated protein kinase
Eif2α: Eukaryotic initiation factor 2 alpha
RECIST: Response Evaluation Criteria in Solid Tumors
